# Report of Deaths in the City of Buffalo for the Month of Sept. 1862

**Published:** 1862-11

**Authors:** Sandford Eastman

**Affiliations:** Health Physician


*Report of Deaths in the City of Buffalo for the month of Sept.* 1862.

Accident 4, Accident by drowning 4, Aneurism 1, Apoplexy, cerebral, 3, Asthma 1, Brain, softening of, 1, Cancer 2, Cancer of the mouth 1, Cancer of the breast 1, Cholera, Asiatic, 1. Cholera infantum 11, Consumption 24, Convulsions 15. Croup 9, Delirium tremens 4, Dentition 5, Diarrhoea 25, Disease of the heart 5, Disease of the liver 3, Diptheria 3, Dropsy of the ovarian 1, Dysentery 6, Epilepsy 1, Fever 1, Fever, scarlet 6, Fever, typhoid 6, Gangrene 1, Hæmorrhage 1, Hæmorrhage from lungs 1, Inflammation of the bowels 2, Inflammation of the brain 5, Inflammation and meninges 4, Inflammation of the lungs 6, Inflammation of the lungs, typhoid 1, Inflammation of the peritoneum 1, Insanity 1, Intemperance 1, Marasmus 4, Measles 1, Necrosis of femur 1, Old age 7, Paralysis I, Premature birth 2, Pyaemia 1, Scrofula 1, Syphilis 1, Tabes mesenterica 2, Thrush infantile 1, Ulcer 1, Unknown 5, Whooping cough 1. Total 197. Still-born 7.

*By whom certified*—By regular physicians at public institutions, 26; by regular physicians in city at large, 65; by irregular practitioners, 39; by coroner, 11; by undertrkers, 63. Total 204.

SANDFORD EASTMAN, Health Physician.

